# Supplementary material for: The Risk of Breast Cancer According to Mutation Type and Position in Carriers of a Pathogenic Variant in BRCA1
Source: Curr Oncol. 2025 Dec 15;32(12):705. doi: 10.3390/curroncol32120705 (PMC12731516; doi:10.3390/curroncol32120705)
Supplement: Supplementary file 1 [file curroncol-32-00705-s001.zip › curroncol-3967955-supplementary.pdf]

SUPPLEMENTAL TABLES AND FIGURES

Table S1. Breast cancer risk among *BRCA1* carriers by exon

Exon	All, n (%)	Breast cancer cases, n (%)	15-year cumulative incidence	HR (95%CI)	*p*	HR (95%CI)[b]	*P*
1	0	0	n/a	n/a			
2	336/3677 (9.1)	36/336 (10.7)	0.23	0.98 (0.52-1.85)	0.95	0.95 (0.50-1.79)	0.87
3	7/3677 (0.2)	0	n/a	0	0.99	0	0.98
4	552/3677 (15.0)	72/552 (13.0)	0.23	0.95 (0.52-1.71)	0.86	1.04 (0.58-1.88)	0.89
5	22/3677 (0.6)	2/22 (9.1)	0.26	0.93 (0.21-4.10)	0.92	0.92 (0.21-4.06)	0.91
6	10/3677 (0.3)	2/10 (20.0)	0.57	2.52 (0.59-11.61)	0.21	2.99 (0.67-13.28)	0.15
7	8/3677 (0.2)	1/8 (12.5)	0.22	1.52 (0.20-11.62)	0.69	1.55 (0.20-11.88)	0.67
8	0	0	n/a	n/a	n/a	n/a	n/a
9	0	0	n/a	n/a	n/a	n/a	n/a
10	935/3677 (25.4)	128/935 (13.7)	0.26	1.12 (0.63-1.99)	0.69	1.15 (0.65-2.03)	0.64
10.1	251/3677 (6.8)	38/251 (15.1)	0.35	1.33 (0.71-2.50)	0.37	1.33 (0.71-2.51)	0.37
10.2	176/3677 (4.8)	21/176 (11.9)	0.20	0.91 (0.45-1.81)	0.78	0.91 (0.45-1.81)	0.78
10.3	166/3677 (4.5)	31/166 (18.7)	0.37	1.69 (0.88-3.22)	0.11	1.70 (0.89-3.24)	0.11
10.4	342/3677 (9.3)	38/342 (11.1)	0.20	0.87 (0.46-1.63)	0.66	0.91 (0.49-1.71)	0.78
11	12/3677 (0.3)	0	n/a	n/a	0.98	0	0.98
12	104/3677 (2.8)	13/104 (12.5)	0.19	1.00 (reference)		1.00 (reference)	
13	14/3677 (0.4)	0	n/a	n/a	0.98		0.98
14	12/3677 (0.3)	1/12 (8.3)	0.22	0.82 (0.11-6.29)	0.85	0.87 (0.11-6.65)	0.89
15	59/3677 (1.6)	8/59 (13.6)	0.36	1.42 (0.59-3.43)	0.44	1.38 (0.57-3.33)	0.47
16	30/3677 (0.8)	8/30 (26.7)	0.50	2.44 (1.01-5.88)	0.05	2.90 (1.20-7.01)	0.02
17	30/3677 (0.8)	4/30 (13.3)	0.25	1.31 (0.43-4.03)	0.63	1.34 (0.44-4.12)	0.61
18	13/3677 (0.4)	0	n/a	n/a	0.98		0.98
19	1476/3677 (40.1)	200/1476 (13.6)	0.25	1.01 (0.57-1.76)	0.98	1.10 (0.63-1.93)	0.74
20	11/3677 (0.3)	2/11 (18.2)	0.29	2.57 (0.58-11.39)	0.21	2.36 (0.53-10.47)	0.26
21	12/3677 (0.3)	1/12 (8.3)	0.09	0.73 (0.10-5.58)	0.76	0.77 (0.10-5.86)	0.80
22	1/3677 (0.03)	0	n/a	n/a	0.99		0.99
23	13/3677 (0.4)	2/13 (15.4)	0.29	1.17 (0.27-5.21)	0.83	1.52 (0.34-6.77)	0.58
**Total[a]**	**3677 (100)**	**481/3677 (13.1)**	**0.25**				

Abbreviations: n/a: not applicable.
[a]Included variants involving more than one exon (n=20) but not included in the counts for individual exons.
[b]Age-adjusted.

Figure S1. Flowchart of study inclusion and exclusion criteria

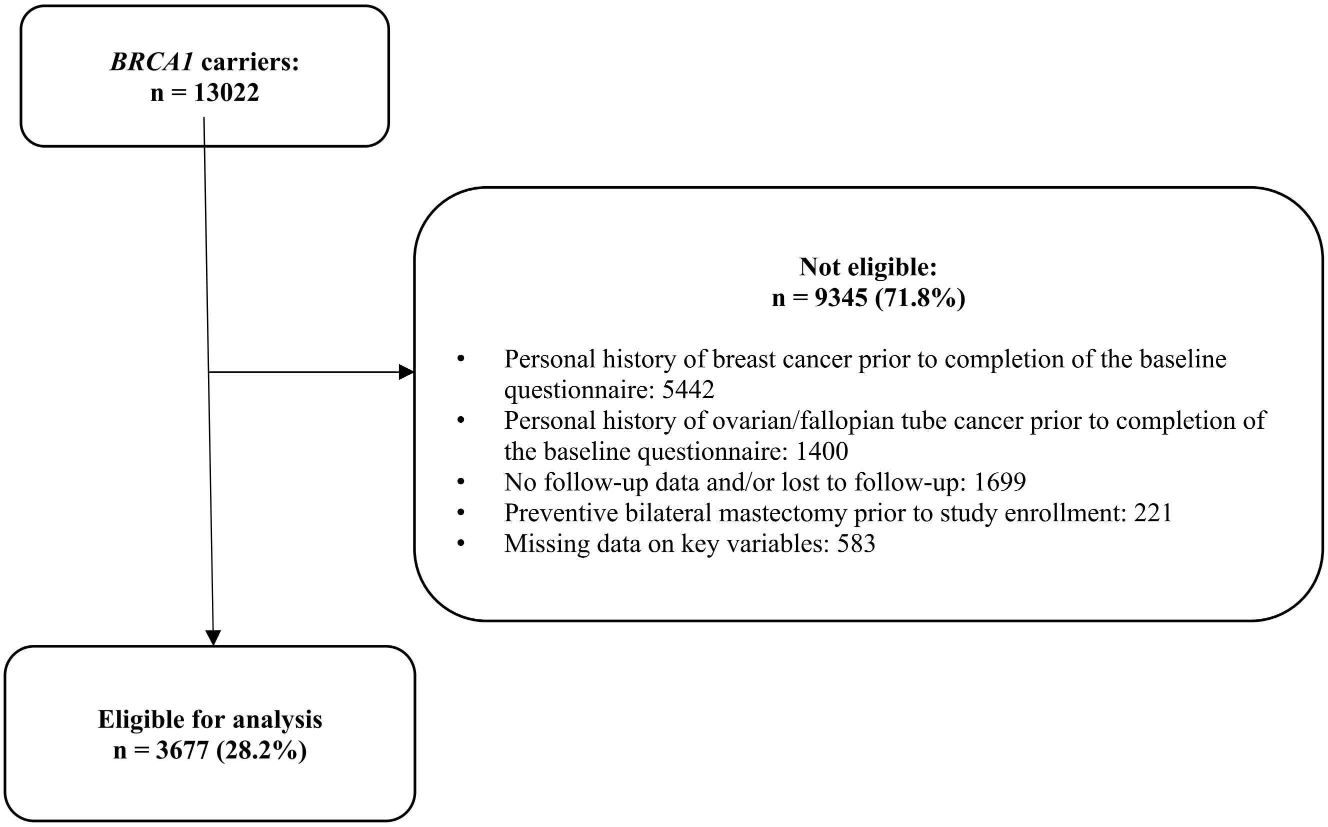

Figure S2. Fifteen-year cumulative risk of breast cancer among *BRCA1* carriers, by exon 10 regions

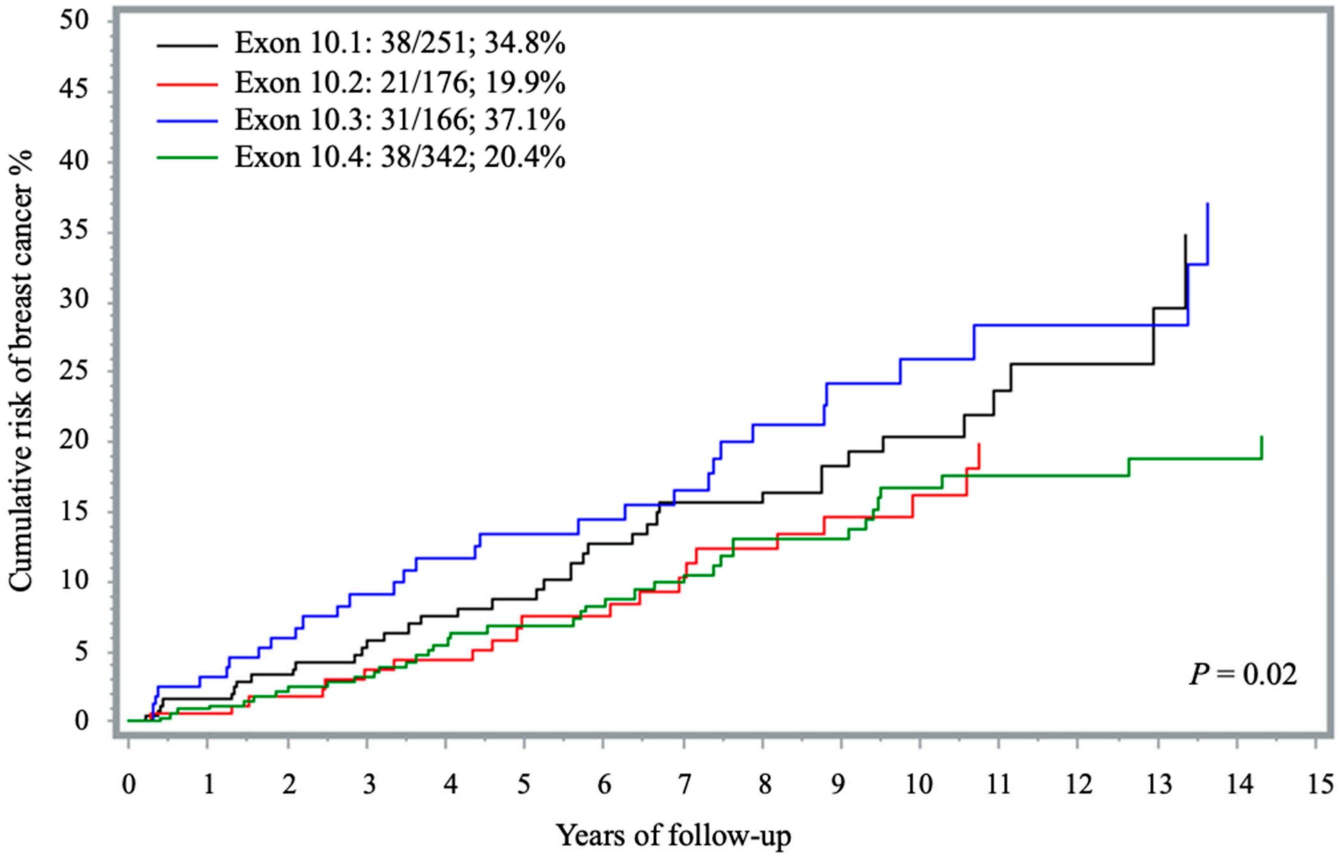

Figure S3. Fifteen-year cumulative risk of breast cancer among *BRCA1* carriers, by NMD status

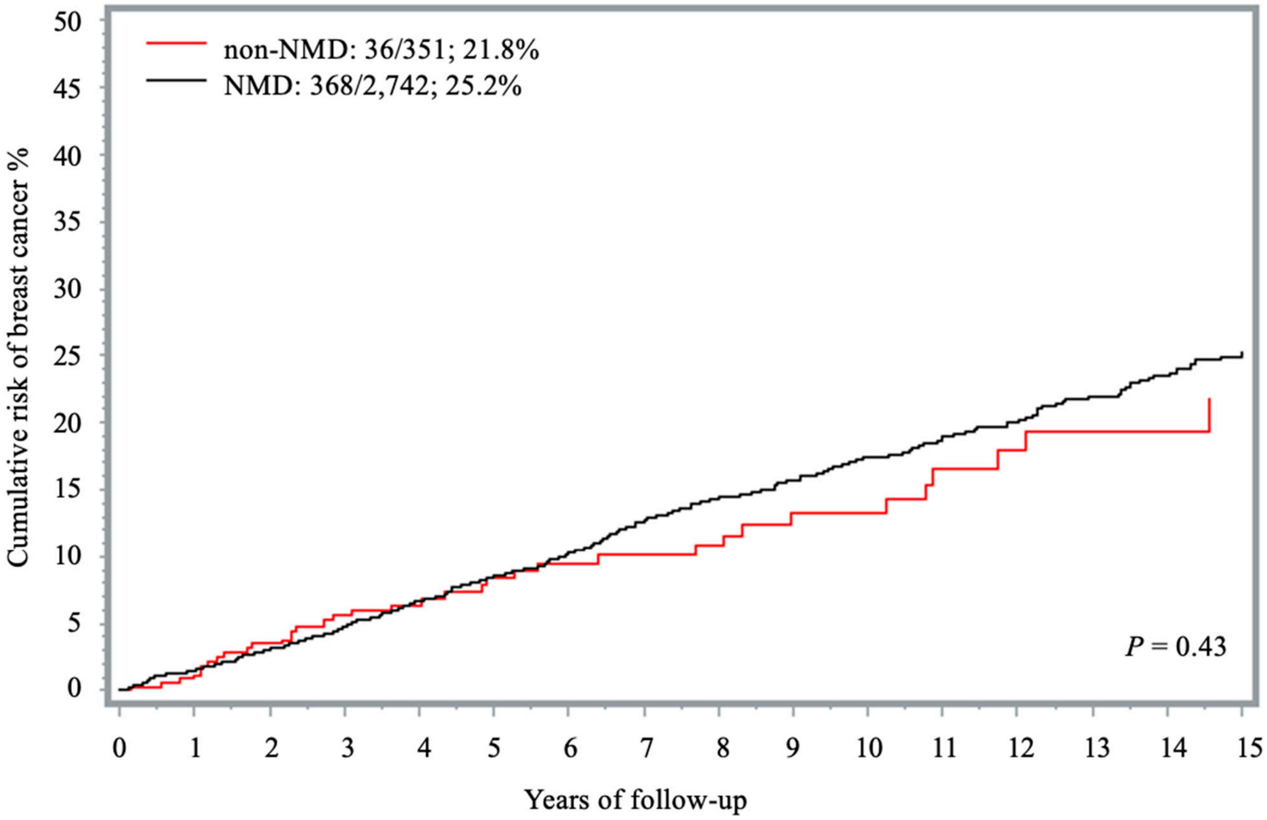

Abbreviations: NMD: Nonsense-mediated decay.

Figure S4. **Fifteen-year cumulative risk of breast cancer among *BRCA1* carriers, by functional class of pathogenic variant (missense vs. truncating)**

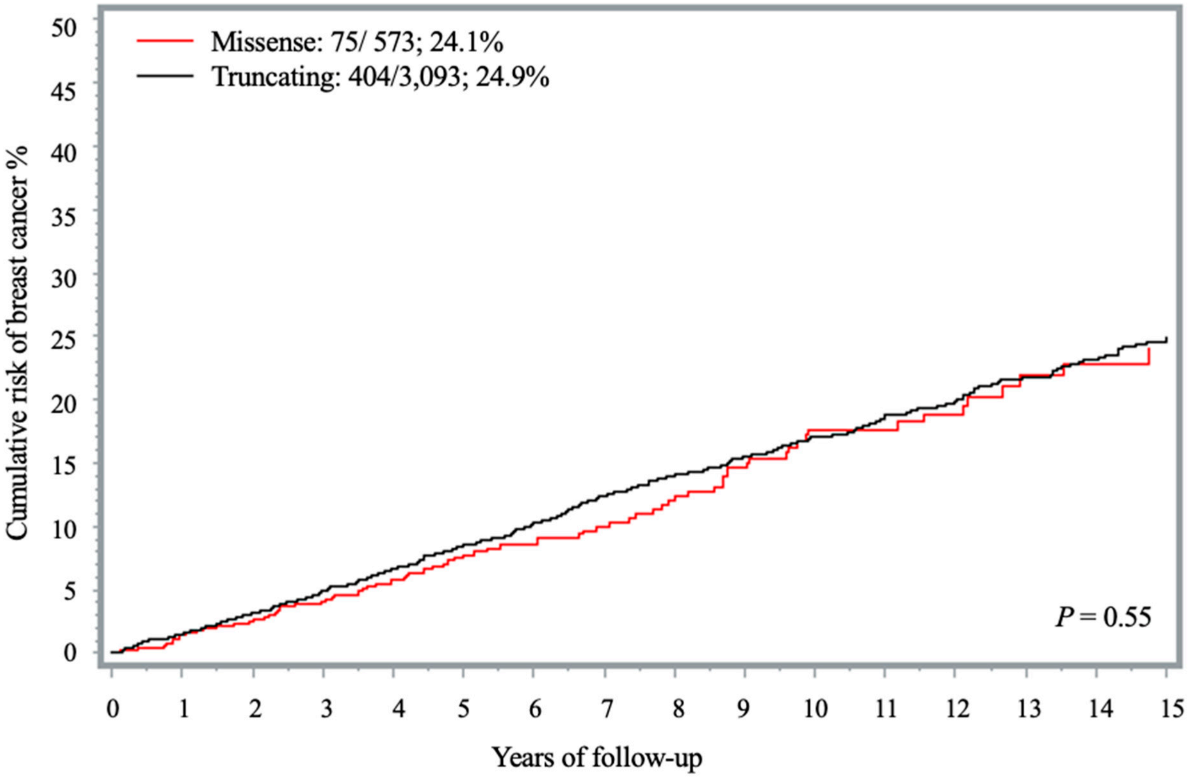

Figure S5. Fifteen-year cumulative risk of breast cancer among *BRCA1* carriers, by cluster region[1]

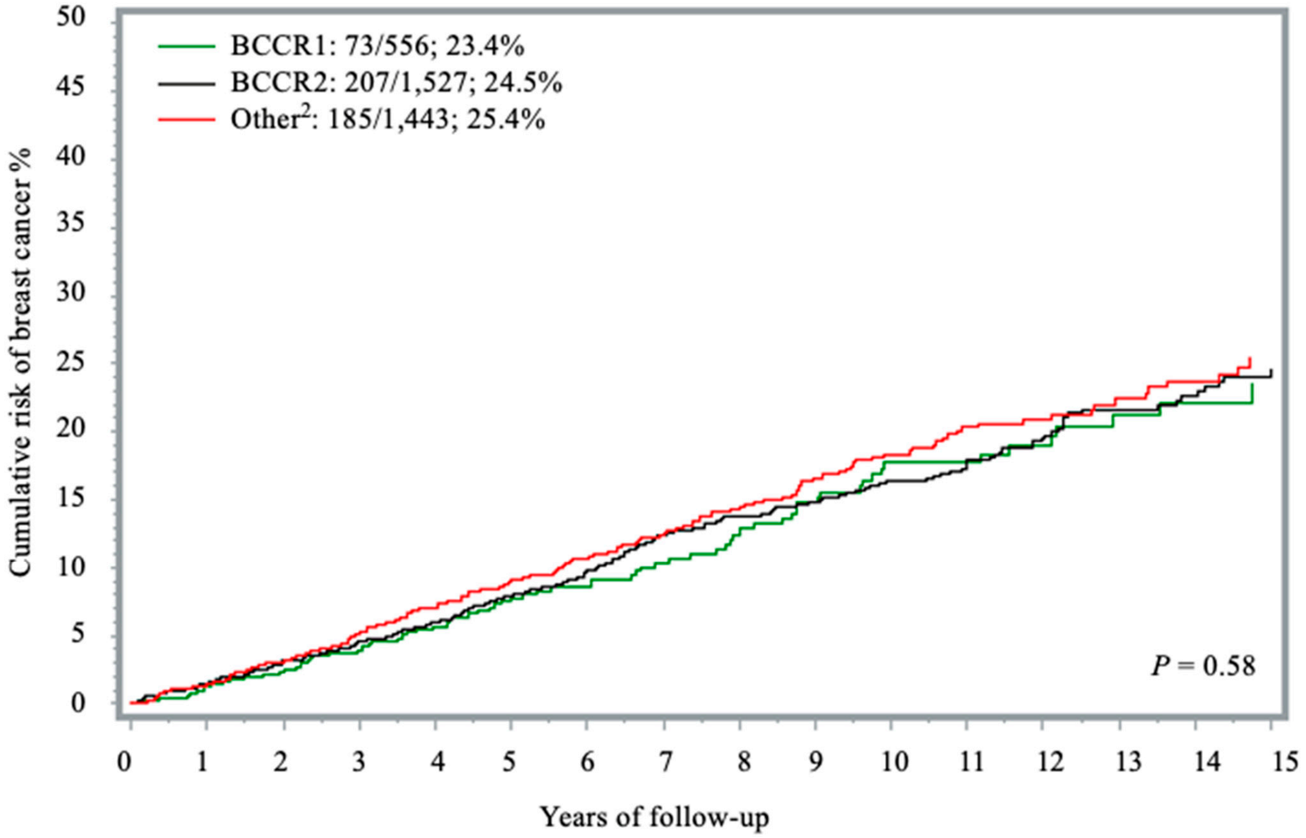

Abbreviations: BCCR, breast cancer cluster region.
[1]Excluded carriers with unknown cluster region mutation (n = 151).
[2]Included mutations involving the ovarian cancer cluster region (OCCR) and mutations outside the BCCRs and OCCR.
